# Epidemiological Prevalence of Phenotypical Resistances and Mobilised Colistin Resistance in Avian Commensal and Pathogenic *E. coli* from Denmark, France, The Netherlands, and the UK

**DOI:** 10.3390/antibiotics11050631

**Published:** 2022-05-07

**Authors:** Andrew Mead, Candice Billon-Lotz, Rikke Olsen, Ben Swift, Pascal Richez, Richard Stabler, Ludovic Pelligand

**Affiliations:** 1 Comparative Biomedical Sciences, Pathobiology and Population Sciences, The Royal Veterinary College (RVC), Hawkshead Lane, Hatfield, Hertfordshire AL9 7TA, UK; bswift@rvc.ac.uk (B.S.); lpelligand@rvc.ac.uk (L.P.); 2 School of Veterinary Medicine, University of Nottingham, Leicestershire LE12 5RD, UK; billonlotz.candice@gmail.com; 3 Department of Veterinary Disease Biology, University of Copenhagen, Stigbøjlen 4, 1870 Frederiksberg C, Denmark; cava@sund.ku.dk; 4 Transpharm, 42 chemin des Olivettes, 34160 Saint-Genies des Mourgues, France; pascal.richez@transpharm.fr; 5 Department of Infection Biology, London School of Hygiene and Tropical Medicine (LSHTM), University of London, London WC1E 7HT, UK; richard.stabler@lshtm.ac.uk

**Keywords:** mobilised colistin resistance (*mcr*), avian pathogenic *E. coli* (APEC), *E. coli*, chicken, epidemiology, colistin

## Abstract

Colistin has been used for the treatment of non-invasive gastrointestinal infections caused by avian pathogenic *E. coli* (APEC). The discovery of mobilised colistin resistance (mcr) in *E. coli* has instigated a One Health approach to minimise colistin use and the spread of resistance. The aim of this study was to compare colistin susceptibility of APECs (collected from Denmark *n* = 25 and France *n* = 39) versus commensal *E. coli* (collected from the Netherlands *n* = 51 and the UK *n* = 60), alongside genetic (*mcr*-1–5) and phenotypic resistance against six other antimicrobial classes (aminoglycosides, cephalosporins, fluoroquinolones, penicillins, sulphonamides/trimethoprim, tetracyclines). Minimum inhibitory concentration (MIC) values were determined using a broth microdilution method (EUCAST guidelines), and phenotypic resistance was determined using disk diffusion. Colistin MIC values of APEC were significantly lower than those for commensals by 1 dilution (*p* < 0.0001, Anderson-Darling test), and differences in distributions were observed between countries. No isolate carried *mcr*-1–5. Three phenotypically resistant isolates were identified in 2/62 APEC and 1/111 commensal isolates. Gentamicin or gentamicin–ceftriaxone co-resistance was observed in two of these isolates. This study showed a low prevalence of phenotypic colistin resistance, with no apparent difference in colistin resistance between commensal *E. coli* strains and APEC strains.

## 1. Introduction

Escherichia coli (*E. coli*) is a commensal organism in the intestinal microbiota of many animals, including chickens [1]. Some strains may cause opportunistic infections, while others contain specific virulence factors that allow them to cause primary disease [2]. Isolates associated with clinical symptoms in birds are often termed avian pathogenic *E. coli* (APEC). Antimicrobial drugs (AMDs), including colistin, have been used to reduce the bacterial burden and risk of disease with colistin entering clinical use shortly after its discovery in 1947 [3]. Colistin has been used for the treatment of non-invasive gastrointestinal infections caused by *E. coli* and APEC, and across Europe, it is approved for administration by drinking water at a dose of 75,000 IU/kg/day for 3–5 days. AMDs are essential components of human and veterinary medicine, but their widespread use has increased the prevalence of antimicrobial resistance (AMR) and, subsequently, has reduced their overall effectiveness [4].

Mobilised colistin resistance (MCR) in livestock *E. coli* has become a significant concern due to the potential for horizontal transfer, following its identification in *E. coli* from pigs [5]. Globally, in both human and animal pathogens, multiple variants have been identified (*mcr*-1 to *mcr*-10), coding for the production of a phosphoethanolamine (PEA) transferase altering the structure of Lipid A and reducing susceptibility to colistin [6,7]. Until recently, when a negative benefit–risk assessment lowered its status, colistin was classified as an antibiotic of critical importance in human medicine due to the increasing incidence of multi-drug-resistant Gram-negative infections. The discovery of MCR led to efforts directed towards reducing its use in veterinary practice. This scrutiny of colistin use, from a One Health perspective, has resulted in a reduction in sales and consumption, in both Europe and several other countries around the world [8].

To limit the selection and spread of low-susceptibility/resistant strains, several surveillance strategies have been put in place at the global, regional, and national levels. Complementary data on sales, use, and resistance contribute to a comprehensive overview of AMR, which is important in identifying trends and providing evidence to advise risk assessment, antimicrobial treatment/stewardship, intervention strategies, and policy while monitoring the impact of these efforts [9]. Phenotypical resistance to colistin is determined by broth microdilution against the epidemiological cut-off (ECOFF), as defined by the European Committee for Antimicrobial Susceptibility Testing (EUCAST; susceptible ≤ 2 mg/L, resistant > 2 mg/L). Current surveillance reports indicate that no colistin-resistant *E. coli* isolates were identified from broiler chickens in 2019 in Denmark, France, the Netherlands, or the UK [10,11,12,13]. However, poultry may act as a reservoir for *E. coli* harbouring *mcr*, with *mcr*-1 being the most prevalent, *mcr*-3 less so, *mcr*-2, 4, and 5 being rarely found and other variants limited to only some bacterial species [14]. Genetic screening for *mcr* genes may provide an addition to standard surveillance methods, although it is not standardised in all surveillance programs and is often reserved for isolates that show phenotypic resistance. Screening only phenotypically resistant isolates may under-represent the prevalence of *mcr*, with several studies demonstrating the presence of *mcr* in phenotypically susceptible *E. coli* [15,16]. Reservoirs of potentially undetected *mcr* in susceptible isolates may still act as a significant reservoir for *mcr* maintenance and spread.

Mobilised colistin resistance has been identified in a range of different plasmids, with *mcr*-1 predominantly found on IncI2 and IncX4 type plasmids [17,18]. Studies have shown that these plasmids may co-carry *mcr*-1 with other resistance genes, including genes encoding resistance to aminoglycosides, β-lactamases (penicillins, cephalosporins, carbapenems), fluoroquinolones, sulphonamides, tetracyclines, and trimethoprim [19,20,21]. Overall, a multi-faceted approach is required to adequately survey the prevalence and spread of colistin resistance and associated resistance genes.

In this study, *E. coli* isolates were received from two pools: (1) from healthy chickens (caecal samples at slaughter from the Netherlands or environmental faecal samples from the UK) and considered as commensal (representative of normal *E. coli* carriage) or (2) from diseased birds (infection with mortality outcome) where the *E. coli* strains obtained were considered as APEC (yolk sac infection from Denmark and the intestinal mucosa from France). The phenotypic and genetic (*mcr*-1–5) resistance of colistin was investigated, alongside phenotypic resistance to six other antimicrobial classes (aminoglycosides, cephalosporins, fluoroquinolones, penicillins, sulphonamides/trimethoprim, and tetracyclines). We hypothesised that (1) distribution of colistin MICs would differ between countries, (2) colistin resistance and presence of *mcr* would be higher in APEC than in commensal *E. coli*, and (3) co-expression of resistance to multiple AMDs would be higher in APEC than in commensal *E. coli*.

## 2. Results

### 2.1. Minimum Inhibitory Concentration (MIC) Distribution of Colistin against E. coli and mcr Status

**Figure 1:**
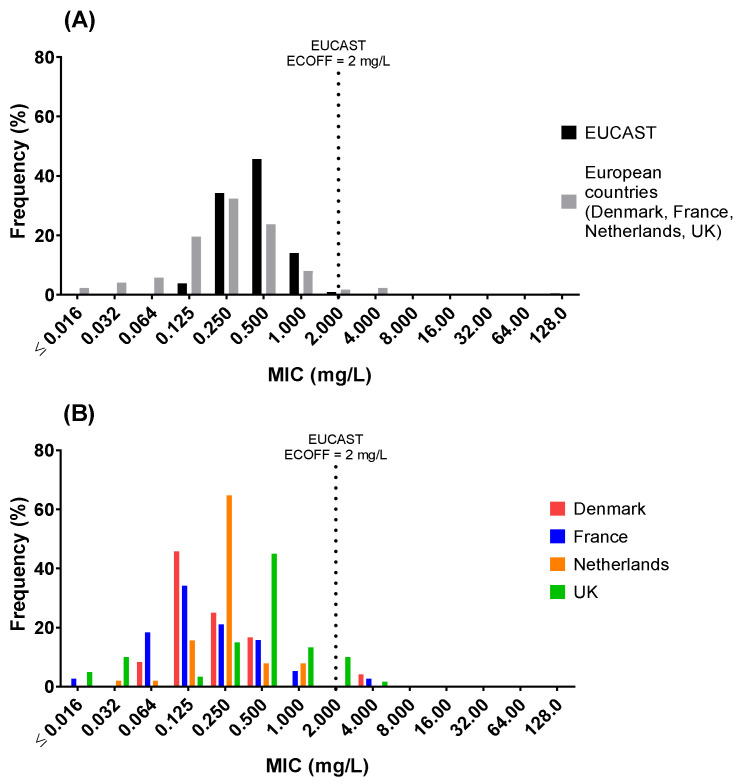
Minimum inhibitory concentration (MIC) distribution for *E. coli* isolates (*n* = 175) collected from Denmark (*n* = 25; avian pathogenic *E. coli* (APEC)), France (*n* = 39; APEC), Netherlands (*n* = 51; commensal) and UK (*n* = 60; commensal) compared to (**A**) the distribution as published by EUCAST (ECOFF = 2 mg/L) and (**B**) between country. Distributions are significantly different (*p* < 0.001), 2-sample Anderson–Darling test.

The distribution of colistin MICs for all poultry isolates was significantly different from the EUCAST distribution (two-sample Anderson–Darling test, *p* ≤ 0.0001), with a mode at 0.25 mg/L vs. EUCAST mode at 0.5 mg/L (Figure 1A), but the 99.5th percentile were similar (2 mg/L). MIC distributions for individual countries (Figure 1B) were as follows: Denmark mode, 0.125 mg/L (range: 0.064–4 mg/L (one isolate—4 mg/L)); France mode, 0.125 mg/L (range: ≤0.016–4 mg/L (one isolate—4 mg/L)); the Netherlands mode, 0.25 mg/L (range: 0.032–1 mg/L); the UK mode, 0.5 mg/L (range: ≤0.016 to 4 (one isolate—4 mg/L)).

**Figure 2:**
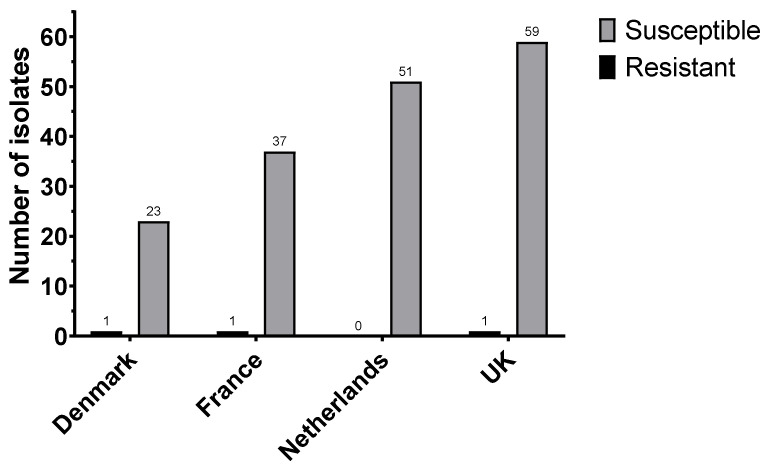
Number of colistin resistant isolates (MIC > ECOFF 2 mg/L) between Denmark (*n* = 1 in 24), France (*n* = 1 in 39), Netherlands (*n* = 0 in 51), and UK (*n* = 1 in 60).

Analysis of the MIC data for all countries showed three less-susceptible/resistant (MIC > 2 mg/L) isolates (one each for Denmark, France, and the UK), indicating a total frequency of 2.3% (Figure 2).

**Figure 3:**
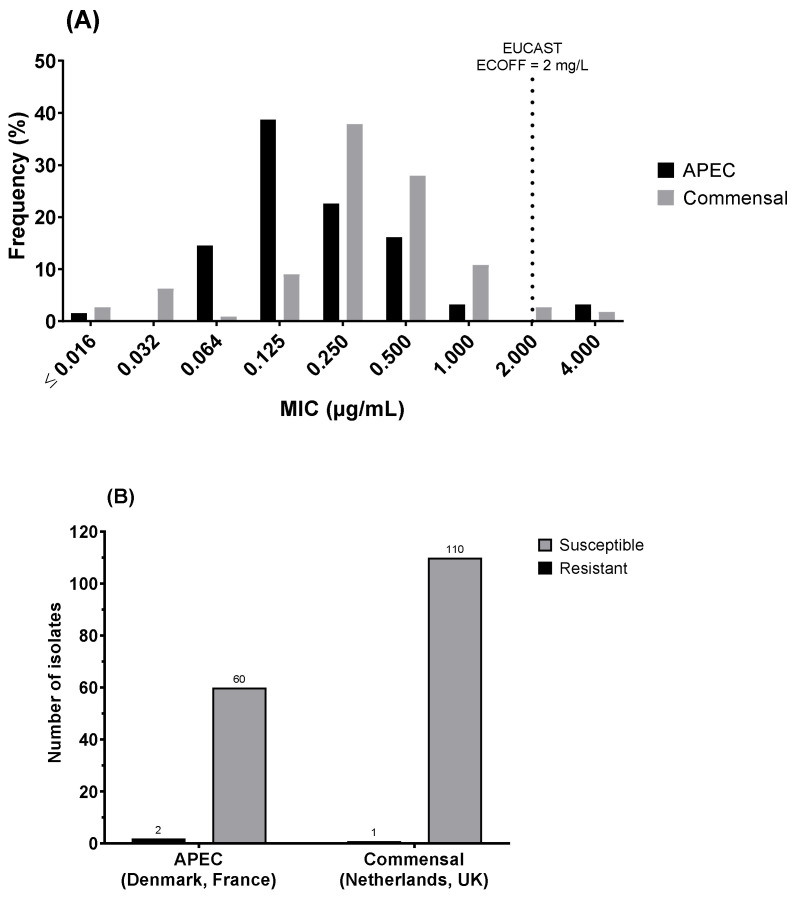
Comparison between colistin susceptibility of avian pathogenic *E. coli* (APEC; Denmark and France) and commensal *E. coli* (Netherlands and UK) through (**A**) the distribution of minimum inhibitory concentration (MIC) and (**B**) the number of resistant isolates (MIC > ECOFF 2mg/L). MIC distributions were significantly different between APEC and commensal *E. coli* (2-sample Anderson–Darling test, *p* < 0.0001), with APEC having a lower mode at 0.125 mg/L), although the relative proportion of resistant isolates was not significantly different between the two groups.

When analysed according to APEC (Denmark and France) vs. commensal (Netherlands and UK) status, a significant difference (two-sample Anderson–Darling test; *p* < 0.0001) was observed in the MIC distributions, with a higher mode at 0.25 mg/L for commensal than for the APEC mode at 0.125 mg/L (Figure 3A). Of the three low-susceptible/resistant isolates, 2 (of 62) were APEC isolates (3.2%), and 1 (of 111) was commensal (0.9%), showing a non-significant difference (Figure 3B).

No isolates tested positive for colistin resistance genes *mcr*-1, 2, 3, 4, or 5.

### 2.2. Phenotypic (Disk Diffusion) Susceptibility to Other AMDs in Conjunction with Colistin Resistance

For the 175 *E. coli* isolates received (APEC *n* = 64: Denmark *n* = 25 (yolk sac infection), France *n* = 39 (clinical infection); commensal *n* = 111: the Netherlands *n* = 51 (caecal sample), the UK *n* = 60 (faecal sample)) phenotypic susceptibility was determined for ampicillin, ceftriaxone, enrofloxacin, gentamicin, tetracycline and trimethoprim/sulfamethoxazole (1:19) with a disk diffusion assay; inhibition diameter distributions are shown in Supplementary Figures S1–S6.

**Figure 4:**
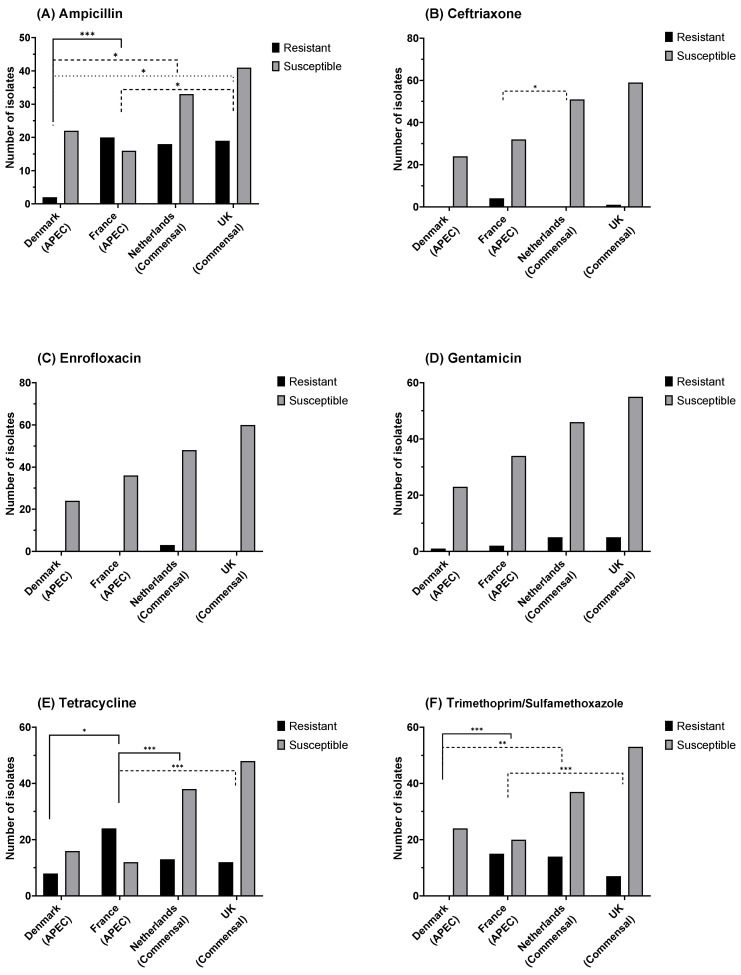
*E. coli* susceptibility as measured by disk diffusion diameter for (**A**) ampicillin (10 µg), (**B**) ceftriaxone (30 µg), (**C**) enrofloxacin (5 µg), (**D**) gentamicin (10 µg), (**E**) tetracycline (30 µg), and (**F**) trimethoprim/sulfamethoxazole (1:19; 25 µg). Fisher’s exact test: * *p* = 0.05, ** *p* = 0.01, *** *p* ≤ 0.001; non-significant differences are not shown.

In total, 60 resistant isolates were identified for ampicillin (Figure 4A), 5 resistant isolates for ceftriaxone (Figure 4B), 3 resistant isolates for enrofloxacin (Figure 4C), 27 resistant isolates for gentamicin (Figure 4D), 57 resistant for tetracycline (Figure 4E), and 29 resistant for trimethoprim/sulfamethoxazole (Figure 4F). Significant differences in the relative frequency of resistant isolates were observed between countries for ampicillin, ceftriaxone, tetracycline, and trimethoprim/sulfamethoxazole.

**Figure 5:**
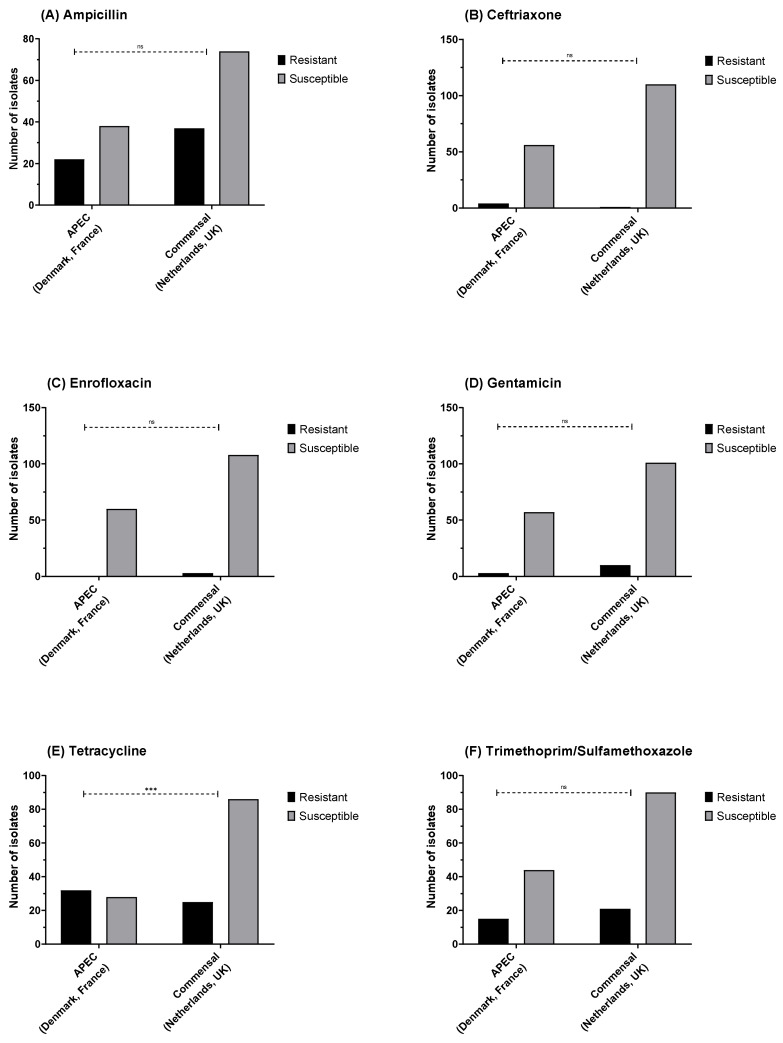
*E. coli* susceptibility as measured by disk diffusion diameter for (**A**) ampicillin (10 µg), (**B**) ceftriaxone (30 µg), (**C**) enrofloxacin (5 µg), (**D**) gentamicin (10 µg), (**E**) tetracycline (30 µg), and (**F**) trimethoprim/sulfamethoxazole (1:19; 25 µg). There was a higher proportion of tetracycline resistance in the avian pathogenic *E. coli* vs. commensals. Fisher’s exact test: *** *p* ≤ 0.001, ns = not significant.

No significant difference was observed in the relative proportions of phenotypically resistant and commensal isolates between APEC and commensal *E. coli* strains, except for tetracycline where the proportion of resistance was higher in APECs (Fisher’s exact test; *p* < 0.001), with 53% resistant in APEC and 22.5% in commensal (Figure 5A–F).

### 2.3. Phenotypic Resistance Patterns and Multi-Drug Resistance (MDR)

**Figure 6:**
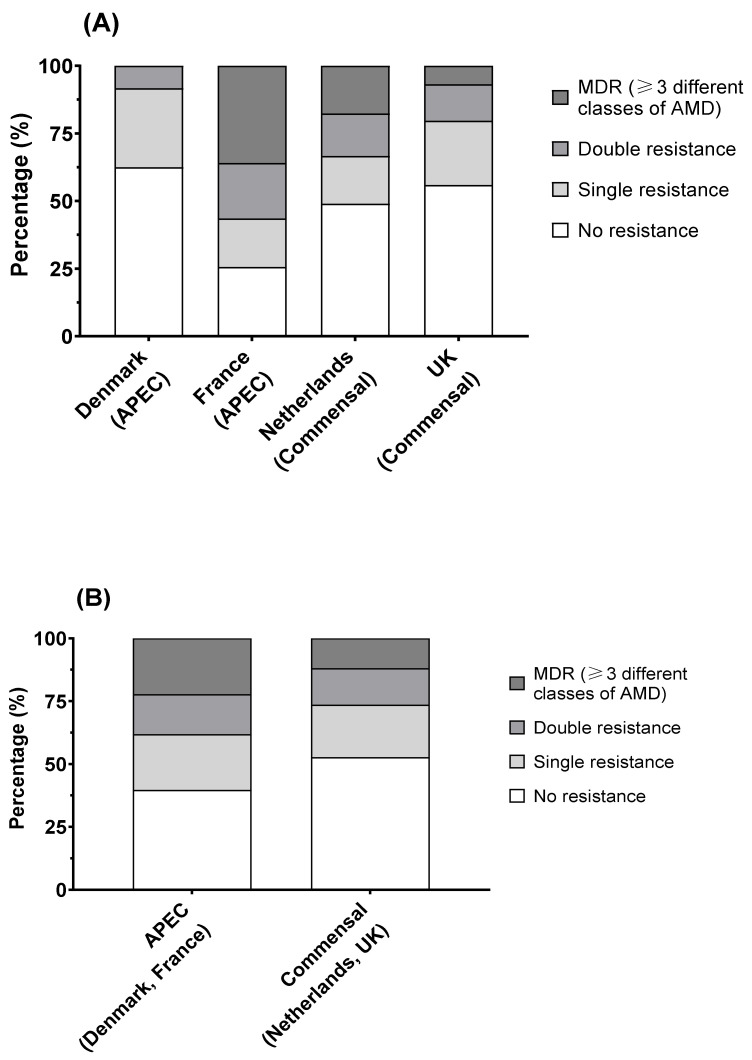
*E. coli* susceptibility as measured by MIC (colistin) and disk diffusion diameter (ampicillin (10 µg), ceftriaxone (30 µg), enrofloxacin (5 µg), gentamicin (10 µg), tetracycline (30 µg), trimethoprim/sulfamethoxazole (1:19; 25 µg)): (**A**) French isolates showed significantly higher proportions of resistant isolates than other countries (Fisher’s exact test; *p* < 0.01), but (**B**) there was no difference between avian pathogenic *E. coli* and commensal *E. coli*.

Overall, Denmark had the highest proportion of isolates showing no resistance (15 of 24; 62.5%), followed by the UK (33 of 60; 55%) and the Netherlands (25 of 51; 49%). France had the lowest proportion of non-resistant isolates at 25.6% (10 of 39). The remaining isolates showed resistance, to a single, double, or multiple (≥3) AMDs. French isolates showed a significant difference (Fisher’s exact test, *p* < 0.001) from the other countries, with higher proportions of isolates showing co-resistance to two antimicrobials (20.5%) and MDR (35.9%), whilst Danish isolates showed the lowest proportion of MDR, at 0% (Figure 6A). No difference was observed in the proportions of isolates in the different groups between APEC and commensal *E. coli* (Figure 6B).

Of the three previously identified colistin-resistant isolates, one isolate (from the UK) showed no phenotypic resistance to any of the other antimicrobials tested. Co-resistance was observed in the remaining two isolates, with the Danish isolate having co-resistance to gentamicin and the French isolate being MDR to colistin, gentamicin, and ceftriaxone. The various resistance patterns are shown in Table 1.

## 3. Discussion

The observed colistin MICs in this study may be considered in line with the EUCAST distribution (ECOFF 2mg/L) with the ECOFF in this study distribution (determined by ECOFFinder) at 2 mg/L, although having a different overall distribution and high variations between countries. Furthermore, the MIC distribution between APEC and commensal isolates was significantly different, with the APEC isolates having a lower mode. APEC is considered a highly diverse population, with studies showing a variety of virulence factors and resistant traits, including tetracyclines, sulphonamides, fluoroquinolones, and polymyxins [2,22]. As such, these disease-causing isolates may be assumed to have a higher phenotypic resistance to colistin than the wild-type commensal isolates, but the lower distribution of MICs for APEC isolates runs counter to this hypothesis.

In this study, a low proportion of phenotypic colistin-resistant *E. coli* isolates was observed in samples obtained from poultry farms in Denmark (4.2%), France (2.63%), the Netherlands (0%), and the UK (1.67%). This study is limited by the small number of isolates tested and the small number of resistant isolates observed, but no significant differences in proportions of colistin-resistant and susceptible isolates were found between countries or between APEC and commensal isolates. Nevertheless, it should be noted that the manner of isolation, e.g., collected from farms, at slaughter, from meat, from faecal material, or (in the case of APEC strains) from infected birds, may affect the number of resistant strains observed. For example, it has been indicated that meat samples have a lower prevalence of colistin resistance than faecal or animal samples [23]. The results of our study, although confounded by different ways of isolation with the APEC isolates from Denmark and France collected from diseased birds, the commensal isolates from the Netherlands obtained from caecal samples at slaughter and the commensal UK isolates from faecal (environmental) samples are consistent with the findings of other studies. Low prevalence of colistin resistance in European countries, taken from faecal or caecal samples, was also shown by Ceccarelli et al. [24] across nine European countries (Belgium, Bulgaria, Denmark, France, Germany, Italy, the Netherlands, Poland, and Spain), Perrin-Guyomard et al. [20] who investigated French isolates, and the surveillance reports for the countries in this study [10,11,12,13].

The low levels of prevalence in Europe, and specifically in the countries within this study, are likely a reflection of the consumption of colistin, in which the use of all antimicrobials for growth promotion purposes was banned in 2003 (European Parliament and Council Regulation 1831/2003/EC). In 2012, it was estimated that 545.2 tonnes of polymyxins (predominantly colistin, as other polymyxins are not approved since there are no minimum residue limits) were consumed by food-producing animals in Europe [25]. In 2015, the EMA reported 561.4 tonnes of polymyxins sold for food-producing animals in 29 European (EU and Switzerland) countries [8]. In 2016, advice from the antimicrobial advice expert group (AMEG) of the EMA prescribed all countries should reduce polymyxin use to prevent the spread of antimicrobial resistance (EMA/CVMP /CHMP/231573/2016). A declining trend in polymyxin usage has resulted in a drop to 210 tonnes by 2018 (31 EU countries median: 1.50 mg/PCU; range: 0 to 12.8 mg/PCU; PCU = population correction unit accounting for animal population and weight), although there was considerable variation between countries [8]. This continued decrease in colistin use has seen polymyxin sales between 2011 and 2018 drop by 69.8%. In 2018 colistin sales, as a percentage of total antimicrobial sales, accounted for <0.1%, 2.8%, 0.65%, and <0.1% for Denmark, France, the Netherlands, and the UK, respectively [8].

For comparison, reports have shown that China has a higher prevalence (14–18%) of colistin resistance in *E. coli* isolated from chicken samples [26,27]. This has been attributed to the fact that China is the largest colistin consumer globally, using between 2470 and 2875 metric tonnes annually (between 2011 and 2015) in animal farming [28]. Following a ban on colistin use as a growth promoter in 2017, reductions in both the production and sale of colistin in China have been recorded, and in line with this decline, a reduction from 18.1% (in 2015–2016) to 5% (in 2017–2018) in the prevalence of colistin-resistant *E. coli* amongst farms was observed [27]. This study also indicated that the co-harbouring of resistance genes for other antimicrobial classes may maintain colistin resistance, even in the absence of colistin use. Similarly, the withdrawal of colistin as a growth promoter in Japan in 2017 resulted in a decrease in plasmid-mediated colistin resistance in pigs by 52.5% in the succeeding 12 months [29]. This indicates a correlation between the use of colistin as a growth promoter and the selection and/or maintenance of colistin-resistant *E. coli* within chicken populations. Likewise, Ahmed et al. [30] demonstrated a positive correlation between the use of colistin in poultry and the frequency of MCR determination. The low consumption in European countries, in combination with strict animal husbandry procedures and regulations, may be a factor in the low prevalence of colistin resistance in poultry *E. coli* observed in this study.

The occurrence of MCR in commensal poultry *E. coli* populations differs significantly between countries in Europe, with 11.9% in Romania [19], 5.7% in Germany, 0.7% in the Netherlands, 0.3% in France, 0% in Denmark, 0% in Italy, and 0% in the UK [31]. In this study, three isolates were identified as being resistant to colistin, although no *mcr* genes were detected amongst all isolates. Luo et al. (2017) explored the prevalence of both chromosomal and plasmid-mediated resistance (47.5% and 52.5%, respectively), showing a similar prevalence between the two mechanisms in colistin-resistant *E. coli*. As no *mcr* genes were identified in this study it is assumed that a chromosomally mediated mechanism is responsible for resistance in these isolates. Previous studies have demonstrated that MCR typically confers a fitness cost on the harbouring bacteria, with *mcr*-1 positive *E. coli* having a lower relative fitness, compared with non-*mcr*-1-expressing *E. coli*, and that overexpression can lead to cell death [32]. Furthermore, a fitness cost is generally associated with resistance carriage and has been demonstrated for other *mcr* genes, coupled with a reduction in virulence [33,34], although this fitness cost may be mediated by other co-mutations [35]. Reduced fitness may reflect the absence of *mcr* genes in samples collected in European countries where the colistin use, and thus selective pressure, is low, and *mcr*-positive *E. coli* are less likely to be maintained in the environment.

In the absence of colistin use, other antimicrobial agents may be used to maintain animal health and welfare. The co-harbouring of resistance genes for other antimicrobials can lead to the continued selection/maintenance of MCR within the population (Wang et al. 2020b). To assess this aspect, our study was extended to investigate other classes of antimicrobials—namely, penicillins (ampicillin), cephalosporins (ceftriaxone), fluoroquinolones (enrofloxacin), aminoglycosides (gentamicin), tetracyclines (tetracycline), and sulphonamides (trimethoprim/sulfamethoxazole)—to identify the occurrence of multi-drug resistance with colistin. A high proportion of isolates were resistant to ampicillin (34.5%), tetracycline (32.7%), and trimethoprim/sulfamethoxazole (17.1%), whilst the proportion of resistance was low for gentamicin (4.09%), ceftriaxone (2.34%), and enrofloxacin (1.75%) in this study. This finding was similarly reported by Roth et al. (2019), except for fluoroquinolones, which were reported with a high prevalence, in broilers in five European countries (Poland, United Kingdom, Germany, France, and Spain). The proportion of resistance correlated with the quantity of antimicrobial consumed, with the highest consumption being reported for penicillins, tetracyclines, sulphonamides, and trimethoprim (ESVAC, 2020). Aminoglycosides also had high consumption, but the proportion of gentamicin resistance was low; this may be attributed to higher use in cattle and pigs compared with poultry. Despite the low resistance to gentamicin in this study, two isolates (one from Denmark and one from France) showed co-resistance for colistin and gentamicin. Several studies have reported co-resistance to colistin and gentamicin in *P. aeruginosa* [36], *K. pneumoniae* [37], and *E. coli* [38] isolated from human samples; in poultry, co-resistance has been reported in *E. coli* [30,39,40]. The selection of colistin–gentamicin co-resistance is often related to the consecutive or concomitant administration of these antimicrobials, such as in cases of cystic fibrosis in man. This may suggest that the use of both colistin and gentamicin in animal populations selects the co-carriage of these resistance genes. In both of the aforementioned examples, *E. coli* isolates were isolated in Bangladesh, and although they were from separate studies, and there was no indication of animal to human spread, the isolation of co-resistance in animal reservoirs requires careful monitoring. Isolates were also identified with co-resistance to tetracycline and ceftriaxone, and as with gentamicin, the presence of isolates co-harbouring resistance may promote the maintenance of these populations in the absence of colistin use where the other antimicrobials continue to be used [41]. In fact, the most common plasmids (IncI2, IncH2, IncX4) carrying *mcr*-1 have also been shown to carry multiple resistance genes, be highly transferable, and confer phenotypic resistance to multiple AMDs including tetracycline, ceftriaxone, and gentamicin [42,43]. Furthermore, *mcr* has been associated with the transposable genetic element ISApl1 in these three plasmids, indicating the potential for transposition of *mcr* to plasmids carrying other resistance elements [44].

The differences in relative proportions of resistant isolates between APEC and commensal *E. coli* presented in this study showed no significant association, likely due to the low sample size. Additionally, the APEC strains originated from Denmark and France, whilst the commensal strains originated from the Netherlands and the UK. This geographical separation may indicate a country-specific effect rather than an APEC–commensal effect, and further study would be required to clarify the origin of this difference, which cannot be identified within the methods reported in this article. Moreover, the distinction between APEC and commensal *E. coli* is questionable. Indeed, a review by Collingwood et al. (2014) states that classifying any disease-causing *E. coli* as APEC is problematic, as a large portion of infection caused by *E. coli* in chickens is opportunistic, meaning a potentially large portion of *E. coli* classified as APEC could in fact be opportunistic commensal *E. coli*. A further definition may be obtained through serotyping isolates or exploring pathogenicity factors associated with APEC.

## 4. Materials and Methods

### 4.1. Sample Origin, Acquisition, and Storage

A total of 175 *E. coli* isolates were received, from which 111 were considered commensals (Netherlands *n* = 51, UK *n* = 60), and 64 were considered avian pathogenic *E. coli* (APEC) (Denmark *n* = 25, France *n* = 39). Danish isolates were collected from chickens suffering from yolk sac infections and provided by Prof. Rikke Olsen and Prof. Peter Damborg (Department of Veterinary and Animal Sciences, Copenhagen University, Cpoenhagen, Denmark). APEC strains from France were isolated from a clinical site of infection via intestinal mucosal scraping and provided by Dr. Pascal Richez (TransPharm, Saint-Genies des Mourgues, France). Commensal *E. coli* from the Netherlands were collected from caeca content of healthy broilers at slaughter and provided by Dr. Kees Veldman (Wageningen Bioveterinary Research, Wageningen University, Wageningen, The Netherlands). UK commensal isolates, sampled from faecal (environmental) samples from broiler chickens at different farms across the UK, were received from Dr. Ben Swift (Department of Pathology and Population Science, Royal Veterinary College, London, UK).

Received isolates were recovered from glycerol storage by overnight incubation in Mueller–Hinton broth (MHB; Oxoid, Basingstoke, UK) at 37 °C, 130 rpm. Overnight cultures were streaked onto MacConkey agar (Oxoid, Basingstoke, UK). After the first MacConkey streak, plates were visually examined to confirm colony morphology representative of *E. coli*, and single colonies were sub-cultured onto Mueller–Hinton agar (MHA; OXOID, UK). All isolates were stored in 25% glycerol:MHB at −80 °C for long-term storage (secondary confirmation as *E. coli* by multiplex PCR of 16s RNA marker during *mcr* screening, see infra).

### 4.2. Minimum Inhibitory Concentration (MIC)

European Pharmacopoeia compliant Meiji Seika Pharma’s Colistin sulphate (ColiMeiji^®^, hereafter ‘colistin’), consisting of 78.53% of a mixture of colistin A (polymyxin E1) and colistin B (polymyxin E2), was supplied by Wyjolab (Chaillac, France). Colistin working stock was prepared at 16 mg/L colistin base immediately prior to use (Appendix A).

Minimum inhibitory concentration (MIC) measurements were performed for all isolates, using the broth microdilution method according to the European Committee for Antimicrobial Testing (EUCAST) guidelines and in accordance with ISO-20776, including two control isolates (*mcr*-1 negative; NCTC 12241 and *mcr*-1 positive; NCTC 13846) (EUCAST, 2020). Final plate concentrations ranged from 0.016 to 8 mg/L.

A two-fold dilution series (0.125–64 mg/L) was prepared in cation-adjusted Mueller–Hinton broth (CAMHB; Oxoid, Basingstoke, UK) using a semi-automated pipetting system (ViaFlo Assist, Integra Biosciences, Thatcham, UK). Bacterial suspensions were prepared from individual colonies suspended in PBS with comparison to 0.5 McFarland standard using Densicheck plus (BioMerieux, Basingstoke, UK). Dilution of this suspension was carried out with CAMHB, to achieve a final, in-plate, inoculum of 5 × 10^5^ CFU/mL, with confirmation of inoculum size performed in parallel. The MIC was recorded following overnight static incubation at 37 °C. Two *E. coli* control isolates were included in each plate. MIC within one dilution of the expected range, to account for possible variation in MIC measurement, confirmed the validity of the results.

### 4.3. Multiplex PCR Screening for mcr

Isolates were screened for the presence of *mcr* genes (*mcr*-1, *mcr*-2, *mcr*-3, *mcr*-4, and *mcr*-5) using a multiplex screening method as described by Rebelo et al. [45]. *E. coli*-specific (16S rRNA) primers described by Le Devendec et al. [46] were used as a control, and as a secondary confirmation for *E. coli* identification, in each reaction. Briefly, the reaction parameters were as follows: The reaction mixture consisted of 12.5 µL DreamTaq green PCR master mix (Fisher Scientific, Basingstoke, UK), 5.5 µL nuclease-free water (Fisher Scientific, UK), 0.5 µL of each of the 12 primers (10 µM) and 2 µL of DNA template. Thermal lysis of 1 mL of overnight culture at 100 °C for 5 min, followed by centrifugation at 16,000× *g*, provided the DNA lysate. The thermal cycler (Techne, UK) conditions were as follows: 15 min denaturation at 94 °C, 25 cycles of 30 s at 94 °C, 90 s at 58 °C, 60 s at 72 °C, and final elongation at 72 °C for 10 min.

PCR amplicons were separated using agarose gel electrophoresis (1.5% agarose; Fisher, UK). Amplicon sizes were determined against GeneRuler 100 bp DNA ladder (Fisher Scientific, UK).

### 4.4. Disk Diffusion Testing for Antimicrobial Susceptibility

Disk diffusion antimicrobial susceptibility testing was performed to assess MDR, as described by EUCAST (EUCAST, 2021). Briefly, 90 mm circular MHA plates were prepared and stored at 4–8 °C for no more than 7 days prior to testing. The inoculum was prepared from a single colony selected from an MHA plate, the same colony used for MIC testing, and resuspended in saline to an optical density equivalent to 0.5 McFarland (Thermo Scientific, Basingstoke, UK). The inoculum was applied to the MHA agar plate and pre-dried within 15 min of preparation, to remove residual moisture, and even coverage over the entire surface area was then ensured. Antimicrobial discs were applied using an antimicrobial susceptibility disk dispenser (Oxoid, Basingstoke, UK) within 15 min of inoculation. Antibiotics tested, representing six different antimicrobial families, were ampicillin, tetracycline, enrofloxacin, ceftriaxone, gentamicin, and trimethoprim/sulfamethoxazole (Oxoid™, UK). Incubation was performed statically at 37 °C overnight (18 ± 2 h). Zone of inhibition was measured using digital callipers (Thermo Scientific, Basingstoke, UK), and classification of susceptibility was determined with EUCAST or CLSI (when data were not available using EUCAST) cut-offs (Appendix B).

### 4.5. Statistical Methods and Analysis

Antimicrobial susceptibility distributions, as determined by MIC for colistin and disk diffusion for ampicillin, tetracycline, enrofloxacin, ceftriaxone, gentamicin, and trimethoprim/sulfamethoxazole, were compared through a 2-sample Anderson–Darling test. Assessment of study distribution was performed using the ECOFFinder [47]. Proportions of susceptible and resistant isolates were compared between countries using Fisher’s exact test.

## 5. Conclusions

In conclusion, although the levels of colistin resistance recorded in this study were low, and no *mcr*-positive isolates were identified, multiple, diverse resistant patterns were observed amongst isolates in this study, including the co-carriage of resistance with colistin. Whether or not the use of colistin may indirectly lead to the selection of other antimicrobial-resistant genes, or vice versa, as well as whether or not the use of other antimicrobials may select or maintain colistin resistance in animal reservoirs in the absence of colistin use remains to be investigated. Although reductions in colistin usage appear to have a positive impact on the prevalence of colistin resistance, phenotypically resistant isolates persist, indicating that chromosomal resistance may provide stable reservoirs of resistance.

The associations between colistin resistance and the APEC vs. commensal status of *E. coli* are not directly apparent; the APEC MIC distributions were actually lower, but the data are confounded by low sample sizes, country-specific differences, and the definition of APEC in sampling.

Overall, colistin use and resistance is a global concern and requires continued surveillance and management from a consortium of countries to maintain low prevalence. The dose regimen for colistin use in food animals should be based on PK/PD criteria, as described in the literature [48], to avoid exposure of the intestinal flora to sub-inhibitory concentrations, likely to select for resistance factors.

## Figures and Tables

**Table 1:** Phenotypic resistance combinations, as measured by broth microdilution (colistin; Col) or disk diffusion (ampicillin; Amp, gentamicin; Gent, tetracycline; Tet, trimethoprim/sulfamethoxazole; Sxt) for poultry *E. coli* isolates from Denmark, France, The Netherlands, and UK.

Resistance Profile	Number of Isolates			
	Denmark	France	Netherlands	UK
No resistance	15 (62.5%)	10 (25.6%)	25 (49%)	33 (55%)
Single resistance	7 (29.2%)	7 (17.9%)	9 (18%)	15 (25%)
Col	-	-	-	1 *
Amp	1	1	2	3
Gent	-	-	4	3
Tet	6	4	3	3
Sxt	-	2	-	-
Double resistance	2 (8.3 %)	8 (20.5%)	8 (15.7%)	7 (11.7%)
Col-Gent	1 *	-	-	-
Amp-Enr	-	-	2	-
Amp-Tet	1	6	1	3
Amp-Sxt	-	1	4	3
Tet-Gent	-	-	-	1
Tet-Sxt	-	1	1	-
MDR resistance (≥3)	0 (0%)	14 (35.9%)	9 (17.6%)	4 (6.7%)
Col-Gent-Ceft	-	1 *	-	-
Amp-Enr-Sxt	-	-	1	-
Amp-Tet-Ceft	-	1	-	-
Amp-Tet-Gent	-	1	-	1
Amp-Tet-Sxt	-	9	7	3
Amp-Tet-Ceft-Sxt	-	1	-	-
Amp-Tet-Enr-Sxt	-	-	-	1
Amp-Tet-Gent-Sxt	-	-	1	-
Tet-Ceft-Sxt	-	1	-	-
Total	24	39	51	60

* Isolates showing phenotypical resistance (MIC > 2 mg/L) to colistin.

## Data Availability

The data presented in this study are available on request from the corresponding author.

## Supplementary Materials

The following supporting information can be downloaded at: https://www.mdpi.com/article/10.3390/antibiotics11050631/s1, Figure S1: *E. coli* Ampicillin (10 µg) disk-diffusion diameter distribution comparison between EUCAST and (A) All tested isolates, (B) Denmark (*n* = 25), (C) France* (*n* = 39), (D) Netherlands (*n* = 51) and (E) UK (*n* = 60). * *p* = 0.05.; Figure S2: *E. coli* Ceftriaxone (30 µg) disk-diffusion diameter distribution comparison between EUCAST and (A) All tested isolates, (B) Denmark*** (*n* = 25), (C) France*** (*n* = 39), (D) Netherlands*** (*n* = 51) and (E) UK*** (*n* = 60). * *p* = 0.05, ** *p* = 0.01, *** *p* ≤ 0.001.; Figure S3: *E. coli* Enrofloxacin (5 µg) disk-diffusion diameter distributions for (A) All tested isolates, (B) Denmark (*n* = 25), (C) France (*n* = 39), (D) Netherlands (*n* = 51) and (E) UK (*n* = 60).; Figure S4: *E. coli* Gentamicin (10 µg) disk-diffusion diameter distribution comparison between EUCAST and (A) All tested isolates, (B) Denmark*** (*n* = 25), (C) France*** (*n* = 39), (D) Netherlands*** (*n* = 51) and (E) UK* (*n* = 60). * *p* = 0.05, ** *p* = 0.01, *** *p* ≤ 0.001.; Figure S5: *E. coli* Tetracycline (30 µg) disk-diffusion diameter distribution comparison between EUCAST and (A) All tested isolates, (B) Denmark*** (*n* = 25), (C) France*** (*n* = 39), (D) Netherlands*** (*n* = 51) and (E) UK*** (*n* = 60). * *p* = 0.05, ** *p* = 0.01, *** *p* ≤ 0.001.; Figure S6: *E. coli* Trimethoprim/Sulfamethoxazole (1:19; 25 µg) disk-diffusion diameter distribution comparison between EUCAST and (A) All tested isolates, (B) Denmark*** (*n* = 25), (C) France (*n* = 39), (D) Netherlands* (*n* = 51) and (E) UK*** (*n* = 60). * *p* = 0.05, ** *p* = 0.01, *** *p* ≤ 0.001.

## Appendix A

To prepare colistin stock, colistin sulphate was weighed (mg) and dissolved in 10 mL of cation-adjusted MHB (CAMHB). Colistin solution was filter-sterilised using a 0.22 µm syringe filter and concentration-adjusted by the proportion of colistin base (78.53%) according to Equation (A1). Colistin solution was then diluted with sterile CAMHB to achieve a final working stock at 16 mg/L colistin base. The stock solution was prepared immediately prior to use.

Equation (A1): Concentration calculation to adjust for the potency of colistin sulphate (* Percentage colistin base calculated from the certificate of analysis for each batch).

(A1) $$\mathrm{Concentration}\;\mathrm{of}\;\mathrm{colistin}\;\mathrm{solution}\;\left(\mathrm{mg}/L\right)=\frac{\mathrm{Mass}\;\mathrm{of}\;\mathrm{colistin}\;\mathrm{sulphate}\;\left(\mathrm{mg}\right)\times {\;\mathrm{Percentage}\;\mathrm{colistin}\;\mathrm{base}\;}^{*}\left(\%\right)}{\mathrm{Volume}\;\mathrm{of}\;\mathrm{CAMHB}\;\left(L\right)}$$

## Appendix B

**Table A1:** Antibiotic, class, concentration (µg), and susceptible cut-off inhibition diameter (mm) as reported by either EUCAST or CLSI.

Antibiotic	Antibiotic Class	Antibiotic Disk Concentration (µg)	Susceptible Cut-Off Value (≥mm)	Guidelines
Ampicillin	Penicillins	10	14	EUCAST
Ceftriaxone	Cephalosporins	30	25	EUCAST
Enrofloxacin	Fluoroquinolones	5	16	CLSI
Gentamicin	Aminoglycosides	10	17	EUCAST
Tetracycline	Tetracyclines	30	11	CLSI
Trimethoprim/Sulfamethoxazole	Diaminopyrimidines/Sulphonamides	25 (1:19)	14	EUCAST

European Committee on Antimicrobial Susceptibility Testing (EUCAST, 2021); Clinical and Laboratory Standards Institute (CLSI, 2013).

## References

[1] Guabiraba R., Schouler C. (2015). Avian colibacillosis: Still many black holes. FEMS Microbiol. Lett..

[2] Nolan L.K., Vaillancourt J.P., Barbieri N.L., Logue C.M. (2020). Colibacillosis. Dis. Poult..

[3] Brownlee G., Jones T. (1948). The polymyxins; A related series of antibiotics derived from B. polymyxa. Biochem. J..

[4] Mendelson M., Matsoso M.P. (2015). The World Health Organization global action plan for antimicrobial resistance. SAMJ S. Afr. Med. J..

[5] Liu Y.-Y., Wang Y., Walsh T.R., Yi L.-X., Zhang R., Spencer J., Doi Y., Tian G., Dong B., Huang X. (2016). Emergence of plasmid-mediated colistin resistance mechanism *MCR*-1 in animals and human beings in China: A microbiological and molecular biological study. Lancet Infect. Dis..

[6] Xu Y., Wei W., Lei S., Lin J., Srinivas S., Feng Y. (2018). An Evolutionarily Conserved Mechanism for Intrinsic and Transferable Polymyxin Resistance. MBio.

[7] Baron S., Hadjadj L., Rolain J.M., Olaitan A.O. (2016). Molecular mechanisms of polymyxin resistance: Knowns and unknowns. Int. J. Antimicrob. Agents.

[8] European Medicines Agency (2020). Sales of Veterinary Antimicrobial Agents in 31 European Countries in 2018.

[9] Sanders P., Vanderhaeghen W., Fertner M., Fuchs K., Obritzhauser W., Agunos A., Carson C., Borck Høg B., Dalhoff Andersen V., Chauvin C. (2020). Monitoring of Farm-Level Antimicrobial Use to Guide Stewardship: Overview of Existing Systems and Analysis of Key Components and Processes. Front. Vet. Sci..

[10] DANMAP (2019). Use of Antimicrobial Agents and Occurrence of Antimicrobial Resistance in Bacteria from Food Animals, Food and Humans in Denmark.

[11] RESAPATH (2021). French Surveillance Network for Antimicrobial Resistance in Diseased Animals, 2019 Annual Report.

[12] Veldman K., Mevius D., Wit B., Pelt W., Franz E., Heederik D. MARAN 2019: Monitoring of antimicrobial resistance and antibiotic usage in animals in the Netherlands in 2018. Combined with NETHMAP-2019: Consumption of antimicrobial agents and antimicrobial resistance among medically important bacteria in the Netherlands, 2019.

[13] UK-VARSS (2019). UK Veterinary Antibiotic Resistance and Sales Surveillance Report (UK-VARSS 2018).

[14] Elbediwi M., Li Y., Paudyal N., Pan H., Li X., Xie S., Rajkovic A., Feng Y., Fang W., Rankin S. (2019). Global burden of colistin-resistant bacteria: Mobilized colistin resistance genes study (1980–2018). Microorganisms.

[15] Teo J.W., Kalisvar M., Venkatachalam I., Ng O.T., Lin R.T., Octavia S. (2018). *mcr*-3 and *mcr*-4 variants in carbapenemase-producing clinical Enterobacteriaceae do not confer phenotypic polymyxin resistance. J. Clin. Microbiol..

[16] Terveer E.M., Nijhuis R.H., Crobach M.J., Knetsch C.W., Veldkamp K.E., Gooskens J., Kuikper E., and Claas E. (2017). Prevalence of colistin resistance gene (*mcr*-1) containing Enterobacteriaceae in feces of patients attending a tertiary care hospital and detection of a *mcr*-1 containing, colistin susceptible *E. coli*. PLoS ONE.

[17] Matamoros S., Van Hattem J.M., Arcilla M.S., Willemse N., Melles D.C., Penders J., Nguyen T., Hoa N., Bootsma M., Genderen P. (2017). Global phylogenetic analysis of *Escherichia coli* and plasmids carrying the *mcr*-1 gene indicates bacterial diversity but plasmid restriction. Sci. Rep..

[18] Wang R., van Dorp L., Shaw L.P., Bradley P., Wang Q., Wang X., Jin L., Zhang Q., Liu Y., Rieux A. (2018). The global distribution and spread of the mobilized colistin resistance gene *mcr*-1. Nat. Commun..

[19] Maciuca I.E., Cummins M.L., Cozma A.P., Rimbu C.M., Guguianu E., Panzaru C., Licker M., Szekely E., Flonta M., Djordjevic S. (2019). Genetic features of *mcr*-1 mediated colistin resistance in CMY-2-producing Escherichia coli from Romanian poultry. Front. Microbiol..

[20] Perrin-Guyomard A., Bruneau M., Houee P., Deleurme K., Legrandois P., Poirier C., Soumet C., Sanders P. (2016). Prevalence of *mcr*-1 in commensal Escherichia coli from French livestock, 2007 to 2014. Eurosurveillance.

[21] Dominguez J.E., Redondo L.M., Figueroa Espinosa R.A., Cejas D., Gutkind G.O., Chacana P.A., Di Conza J., Miyakawa M. (2018). Simultaneous carriage of *mcr*-1 and other antimicrobial resistance determinants in *Escherichia coli* from poultry. Front. Microbiol..

[22] Thomrongsuwannakij T., Blackall P.J., Djordjevic S.P., Cummins M.L., Chansiripornchai N. (2020). A comparison of virulence genes, antimicrobial resistance profiles and genetic diversity of avian pathogenic Escherichia coli (APEC) isolates from broilers and broiler breeders in Thailand and Australia. Avian Pathol..

[23] Irrgang A., Roschanski N., Tenhagen B.-A., Grobbel M., Skladnikiewicz-Ziemer T., Thomas K., Roesler U., Kasbohrer A. (2016). Prevalence of *mcr*-1 in *E. coli* from livestock and food in Germany, 2010–2015. PLoS ONE.

[24] Ceccarelli D., Hesp A., Van Der Goot J., Joosten P., Sarrazin S., Wagenaar J.A. (2020). Antimicrobial resistance prevalence in commensal Escherichia coli from broilers, fattening turkeys, fattening pigs and veal calves in European countries and association with antimicrobial usage at country level. J. Med. Microbiol..

[25] Webb H.E., Angulo F.J., Granier S.A., Scott H.M., Loneragan G.H. (2017). Illustrative examples of probable transfer of resistance determinants from food animals to humans: Streptothricins, glycopeptides, and colistin. F1000Res.

[26] Huang X., Yu L., Chen X., Zhi C., Yao X., Liu Y., Wu S., Guo Z., Yi Z., Zeng Z. (2017). High prevalence of colistin resistance and *mcr*-1 gene in Escherichia coli isolated from food animals in China. Front. Microbiol..

[27] Wang Y., Xu C., Zhang R., Chen Y., Shen Y., Hu F., Liu D., Lu J., Guo Y., Xia X. (2020). Changes in colistin resistance and *mcr*-1 abundance in Escherichia coli of animal and human origins following the ban of colistin-positive additives in China: An epidemiological comparative study. Lancet Infect. Dis..

[28] Shen Z., Wang Y., Shen Y., Shen J., Wu C. (2016). Early emergence of *mcr*-1 in Escherichia coli from food-producing animals. Lancet Infect. Dis..

[29] Makita K., Fujimoto Y., Sugahara N., Miyama T., Usui M., Asai T., Kawanishi M., Ozawa M., Tamura Y. (2020). Quantitative release assessment of *mcr*-mediated colistin-resistant Escherichia coli from Japanese pigs. Food Saf..

[30] Ahmed S., Das T., Islam M.Z., Herrero-Fresno A., Biswas P.K., Olsen J.E. (2020). High prevalence of *mcr*-1-encoded colistin resistance in commensal Escherichia coli from broiler chicken in Bangladesh. Sci. Rep..

[31] El Garch F., de Jong A., Bertrand X., Hocquet D., Sauget M. (2018). *mcr*-1-like detection in commensal Escherichia coli and Salmonella spp. from food-producing animals at slaughter in Europe. Vet. Microbiol.

[32] Yang Q., Li M., Spiller O.B., Andrey D.O., Hinchliffe P., Li H., MacLean C., Niumsup P., Powell L., Pritchard M. (2017). Balancing *mcr*-1 expression and bacterial survival is a delicate equilibrium between essential cellular defence mechanisms. Nat. Commun..

[33] Bengtsson-Palme J., Jonsson V., Hess S. (2021). What is the role of the environment in the emergence of novel antibiotic resistance genes?—A modelling approach. Environ. Sci. Technol..

[34] Li H., Wang Y., Chen Q., Xia X., Shen J., Wang Y., Shao B. (2021). Identification of Functional Interactome of Colistin Resistance Protein *MCR*-1 in *Escherichia coli*. Front. Microbiol..

[35] Yang Q.E., MacLean C., Papkou A., Pritchard M., Powell L., Thomas D., Andrey D., Li M., Spiller B., Yang W. (2020). Compensatory mutations modulate the competitiveness and dynamics of plasmid-mediated colistin resistance in *Escherichia coli* clones. ISME J..

[36] Pitt T., Sparrow M., Warner M., Stefanidou M. (2003). Survey of resistance of *Pseudomonas aeruginosa* from UK patients with cystic fibrosis to six commonly prescribed antimicrobial agents. Thorax.

[37] Lübbert C., Faucheux S., Becker-Rux D., Laudi S., Dürrbeck A., Busch T., Gastmeier P., Eckmanns T., Rodloff A., Kaisers U. (2013). Rapid emergence of secondary resistance to gentamicin and colistin following selective digestive decontamination in patients with KPC-2-producing Klebsiella pneumoniae: A single-centre experience. Int. J. Antimicrob. Agents.

[38] Johura F.-T., Tasnim J., Barman I., Biswas S.R., Jubyda F.T., Sultana M., George C., Camilli A., Seed K., Ahmed N. (2020). Colistin-resistant Escherichia coli carrying *mcr*-1 in food, water, hand rinse, and healthy human gut in Bangladesh. Gut Pathog..

[39] Nguyen N.T., Nguyen H.M., Nguyen C.V., Nguyen T.V., Nguyen M.T., Thai H.Q., Ho M.H., Thwaites G., Ngo H.T., Baker S. (2016). Use of Colistin and Other Critical Antimicrobials on Pig and Chicken Farms in Southern Vietnam and Its Association with Resistance in Commensal Escherichia coli Bacteria. Appl. Environ. Microbiol..

[40] Corvec S., Furustrand Tafin U., Betrisey B., Borens O., Trampuz A. (2013). Activities of fosfomycin, tigecycline, colistin, and gentamicin against extended-spectrum-β-lactamase-producing *Escherichia coli* in a foreign-body infection model. Antimicrob. Agents Chemother..

[41] Cao Y.-P., Lin Q.-Q., He W.-Y., Wang J., Yi M.-Y., Lv L.-C., Yang J., Liu J.-H., Guo J.-Y. (2020). Co-selection may explain the unexpectedly high prevalence of plasmid-mediated colistin resistance gene *mcr*-1 in a Chinese broiler farm. Zool. Res..

[42] Migura-Garcia L., González-López J.J., Martinez-Urtaza J., Aguirre Sánchez J., Moreno-Mingorance A., Perez de Rozas A., Hofle U., Ramiro Y., Gonzalez-Escalona N. (2020). mcr-colistin resistance genes mobilized by IncX4, IncHI2, and IncI2 plasmids in Escherichia coli of pigs and White Stork in Spain. Front. Microbiol..

[43] Zhang S., Huang Y., Yang G., Lei T., Chen M., Ye Q., Wang J., Gu Q., Wei X., Zhang J. (2021). High prevalence of multidrug-resistant *Escherichia coli* and first detection of IncHI2/IncX4-plasmid carrying *mcr*-1 *E. coli* in retail ready-to-eat foods in China. Int. J. Food Microbiol..

[44] Snesrud E., He S., Chandler M., Dekker J.P., Hickman A.B., McGann P., Dyda F. (2016). A model for transposition of the colistin resistance gene *mcr*-1 by IS*Apl1*. Antimicrob. Agents Chemother..

[45] Rebelo A.R., Bortolaia V., Kjeldgaard J.S., Pedersen S.K., Leekitcharoenphon P., Hansen I.M., Guerra B., Malorny B., Borowiak M., Hammerl J. (2018). Multiplex PCR for detection of plasmid-mediated colistin resistance determinants, *mcr*-1, *mcr*-2, *mcr*-3, *mcr*-4 and *mcr*-5 for surveillance purposes. Eurosurveillance.

[46] Le Devendec L., Mourand G., Bougeard S., Leaustic J., Jouy E., Keita A., Couet W., Rousset N., Kempf I. (2016). Impact of colistin sulfate treatment of broilers on the presence of resistant bacteria and resistance genes in stored or composted manure. Vet. Microbiol..

[47] Turnidge J., Kahlmeter G., Kronvall G. (2006). Statistical characterisation of bacterial wild-type MIC value distributions and the determination of epidemiological cut-off values. Clin. Microbiol. Infect..

[48] Mead A., Richez P., Azzariti S., Pelligand L. (2021). Pharmacokinetics of Colistin in the Gastrointestinal Tract of Poultry Following Dosing via Drinking Water and Its Bactericidal Impact on Enteric Escherichia coli. Front. Vet. Sci..

