# Supplementary material for: Epidemiological Prevalence of Phenotypical Resistances and Mobilised Colistin Resistance in Avian Commensal and Pathogenic E. coli from Denmark, France, The Netherlands, and the UK
Source: Antibiotics (Basel). 2022 May 7;11(5):631. doi: 10.3390/antibiotics11050631 (PMC9137498; doi:10.3390/antibiotics11050631)
Supplement: Supplementary file 1 [file antibiotics-11-00631-s001.zip › antibiotics-1690141-supplementary.pdf]

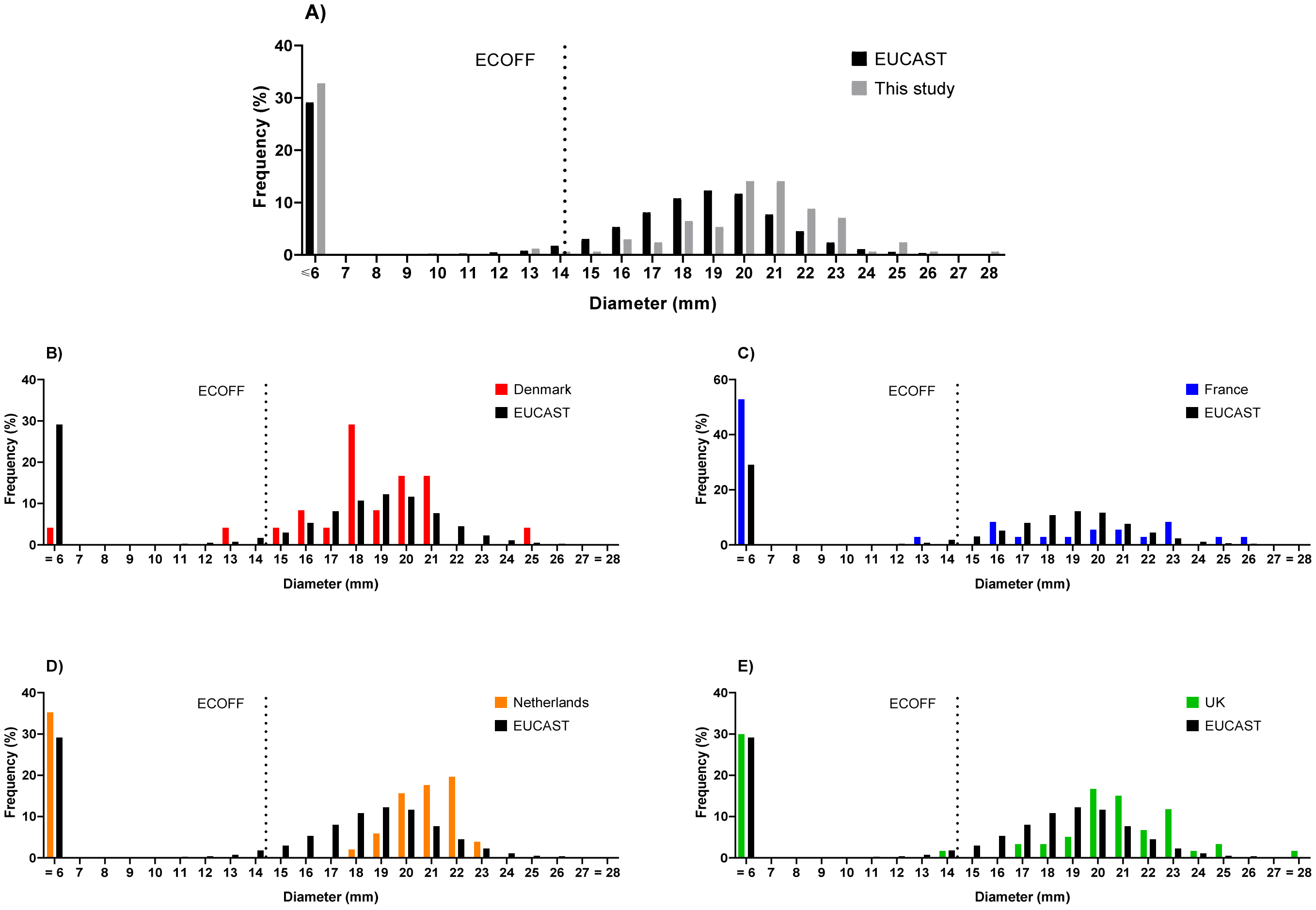

Supplementary Figure S1. *E. coli* ampicillin (10 μg) disk-diffusion diameter distribution comparison between EUCAST and (A) all tested isolates, (B) Denmark (n = 25), (C) France* (n = 39), (D) Netherlands (n = 51), and (E) UK (n = 60). * p = 0.05.

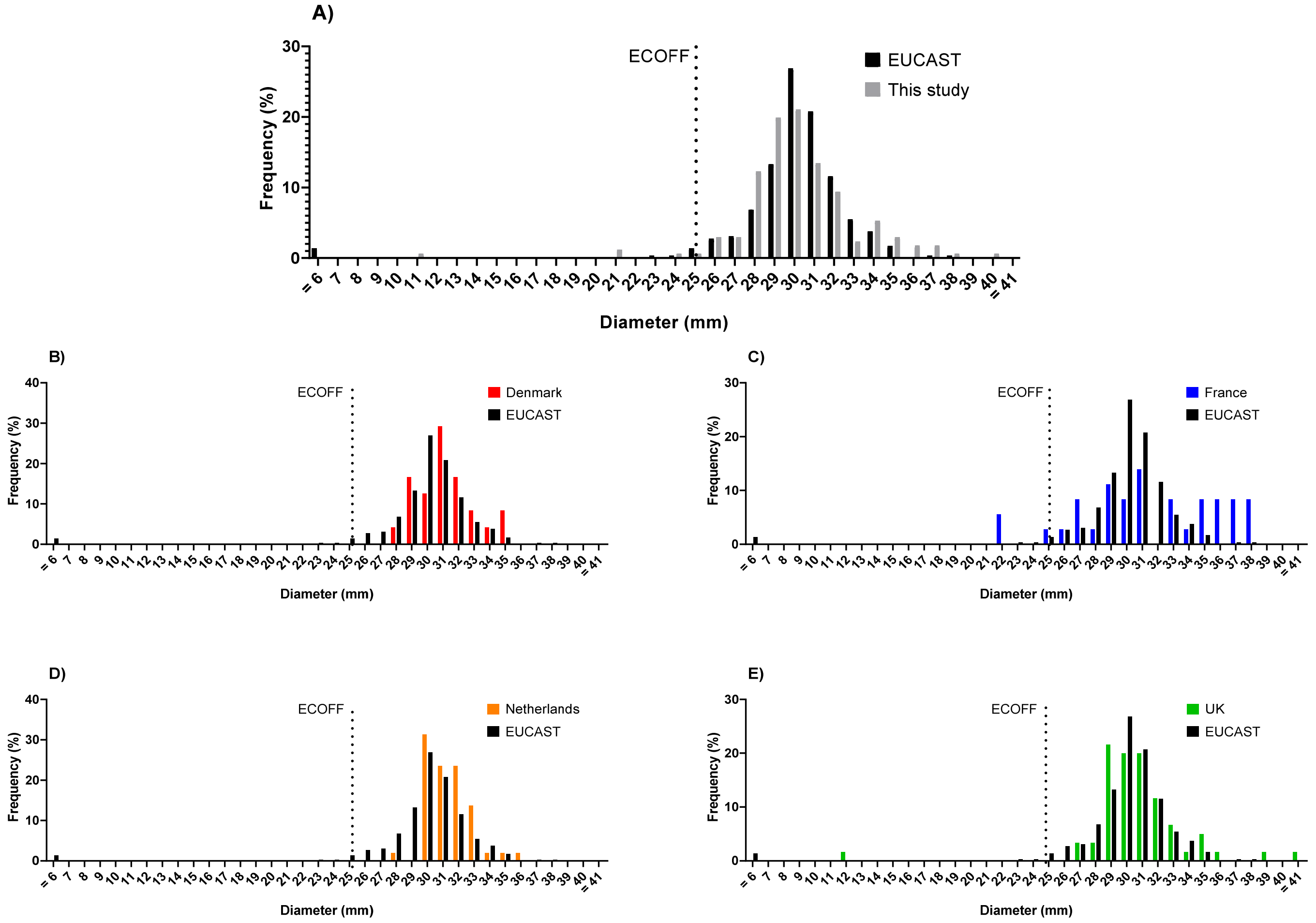

Supplementary Figure S2. *E. coli* Ceftriaxone (30 µg) disk-diffusion diameter distribution comparison between EUCAST and (A) all tested isolates, (B) Denmark*** (n = 25), (C) France*** (n = 39), (D) Netherlands*** (n = 51), and (E) UK*** (n = 60). * p = 0.05, ** p = 0.01, *** p ≤ 0.001.

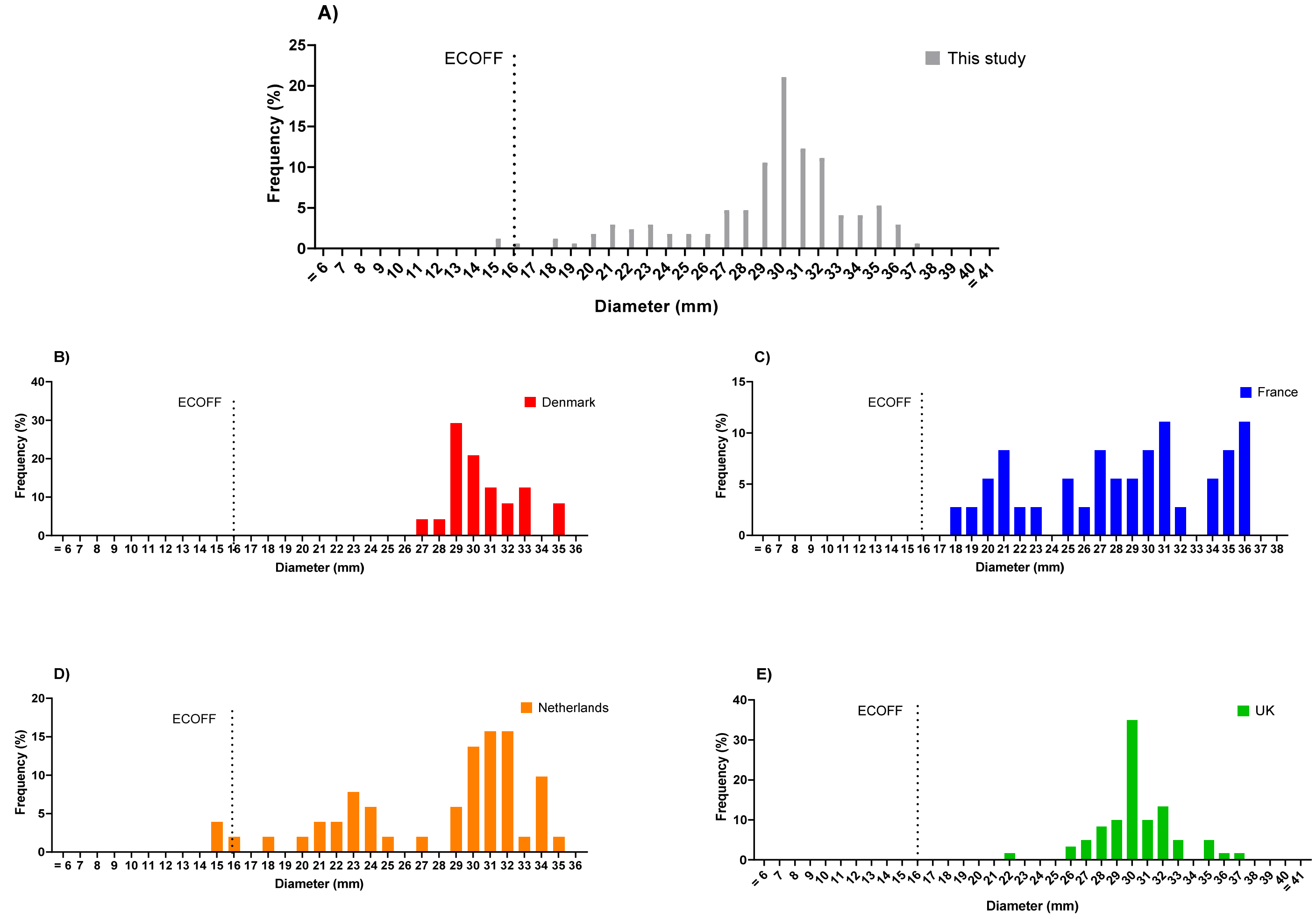

Supplementary Figure S3. *E. coli* Enrofloxacin (5 μg) disk-diffusion diameter distributions for (A) all tested isolates, (B) Denmark (n = 25), (C) France (n = 39), (D) Netherlands (n = 51), and (E) UK (n = 60).

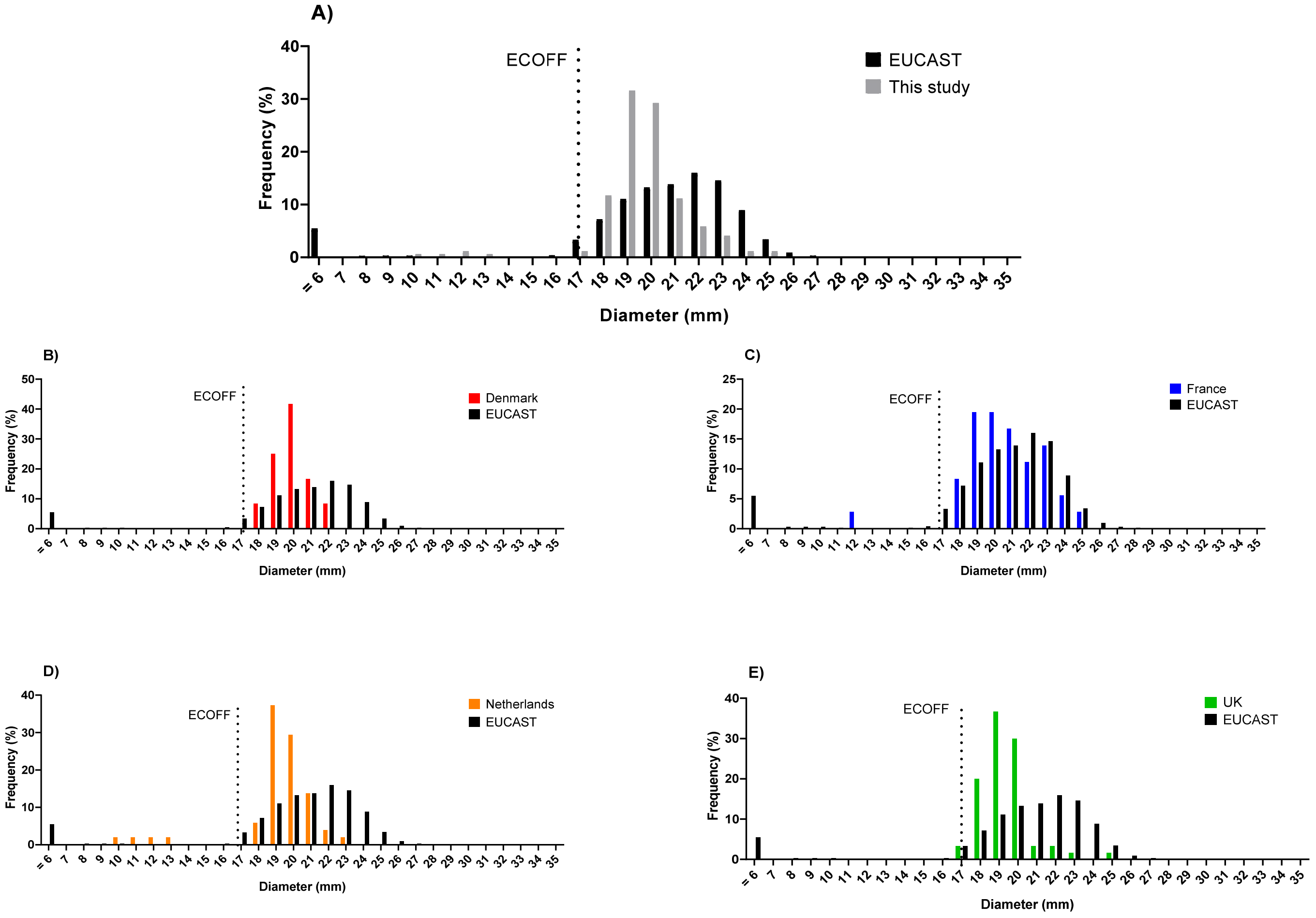

Supplementary Figure S4. *E. coli* Gentamicin (10 µg) disk-diffusion diameter distribution comparison between EUCAST and (A) all tested isolates, (B) Denmark*** (n = 25), (C) France*** (n = 39), (D) Netherlands*** (n = 51), and (E) UK* (n = 60). * p = 0.05, ** p = 0.01, *** p ≤ 0.001.

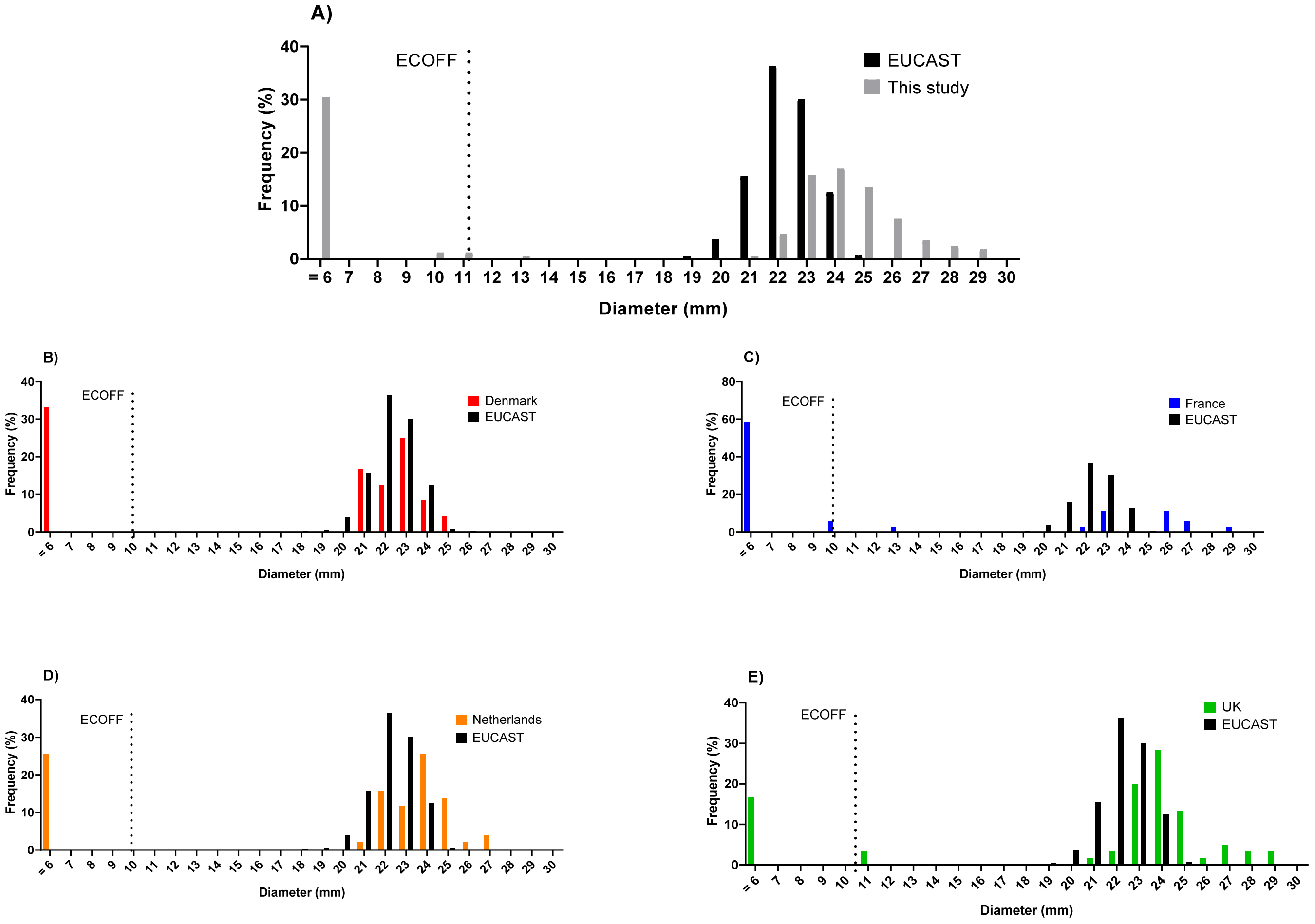

Supplementary Figure S5. *E. coli* Tetracycline (30 µg) disk-diffusion diameter distribution comparison between EUCAST and (A) all tested isolates, (B) Denmark*** (n = 25), (C) France*** (n = 39), (D) Netherlands*** (n = 51), and (E) UK*** (n = 60). * p = 0.05, ** p = 0.01, *** p ≤ 0.001.

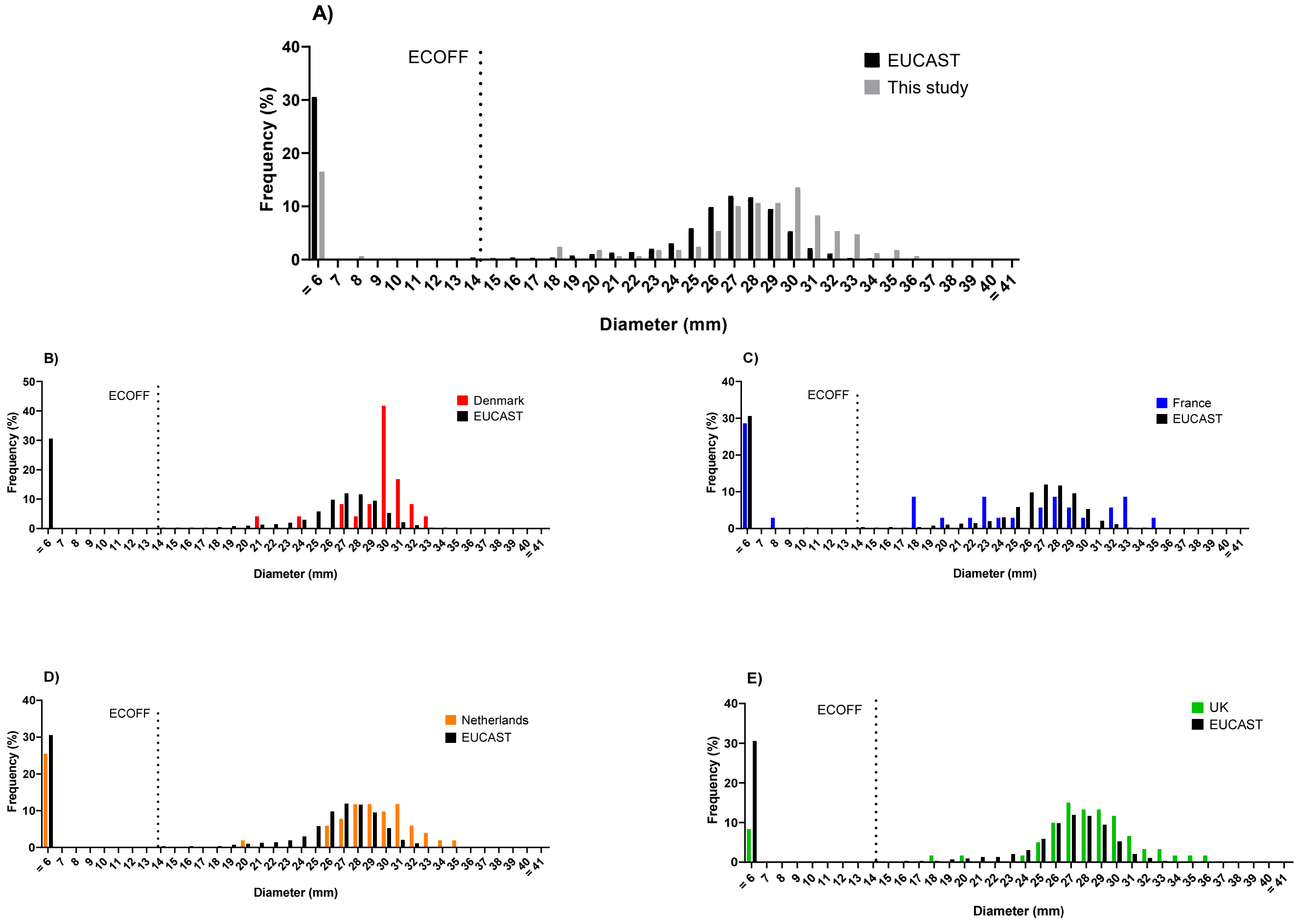

Supplementary Figure S6. *E. coli* Trimethoprim/Sulfamethoxazole (1:19; 25 µg) disk-diffusion diameter distribution comparison between EUCAST and (A) all tested isolates, (B) Denmark*** (n = 25), (C) France (n = 39), (D) Netherlands* (n = 51), and (E) UK*** (n = 60). * p = 0.05, ** p = 0.01, *** p ≤ 0.001.
